# Efficacy of Artesunate + Sulphadoxine-Pyrimethamine (AS + SP) and Amodiaquine + Sulphadoxine-Pyrimethamine (AQ + SP) for Uncomplicated *falciparum* Malaria in Equatorial Guinea (Central Africa)

**DOI:** 10.1155/2009/781865

**Published:** 2009-05-27

**Authors:** Pilar Charle, Pedro Berzosa, Miguel Angel Descalzo, Aida de Lucio, Jose Raso, Jacqueline Obono, Magdalena Lwanga, Natividad Nlang, Araceli Nchama, Catalina Mangue, Anastasio Micha, Natividad Nsee, Rosario Mesie, Agustín Benito, Jesús Roche

**Affiliations:** ^1^Centre for the Control of Diseases, Reference Laboratory of Malaria, National Centre of Tropical Medicine—ISCIII, Malabo, Equatorial Guinea; ^2^National Centre of Tropical Medicine, Institute of Health Carlos III, 28029 Madrid, Spain; ^3^Reference Laboratory of Malaria, MINSABS and ISCIII, Malabo, Equatorial Guinea; ^4^Reference Laboratory of Malaria, MINSABS and ISCIII, Bata, Equatorial Guinea; ^5^Paediatric Department, Regional Hospital of Malabo, Malabo, Equatorial Guinea

## Abstract

*Objectives*. The objectives of the study were (i) to evaluate the efficacy of combination drugs, such as artesunate + sulphadoxine-pyrimethamine (AS + SP) and amodiaquine + sulphadoxine-pyripethamine (AQ + SP) in treatment of uncomplicated *falciparum* malaria (ii) to differentiate recrudescence from reinfection by analysing *msp-1* and *msp-2* genes of *Plasmodium falciparum* in treatment failure cases. *Methods*. We carried out an in vivo study in the year 2005 in 206 children between 6 to 59 months age groups. Of the 206, 120 received AQ + SP, and 86 received AS + SP. A clinical and parasitological followup during 14 days was undertaken. Finger-prick blood sample from each patient was taken on Whatman filter paper (no. 3) on days 0, 7, 14 and also the day when the parasite and symptoms reappeared for PCR analysis. *Results*. Late treatment failure was observed in 3.5% (4/114) with AQ + SP, and 2.5% (2/79) with AS + SP. The success rate was 96.5% with AQ + SP and 97.5% with AS + SP. No deaths and severe reactions were recorded. Out of the 6 treatment failure cases, one was reinfection as observed by PCR analysis of *msp*-1 and *msp*-2 genes on day 14. *Discussion*. Both the combinations found to be efficacious and safe and could be used as a first-line treatment for uncomplicated *falciparum* malaria in Equatorial Guinea.

## 1.Background

Malaria is the major cause of morbidity and mortality in children under five years in Sub-Saharan Africa [1]. In areas of stable malaria transmission, like Equatorial Guinea, 25–40% of the patients in clinics and 20–50% of hospital admissions are due to malaria. The *falciparum* malaria is the cause of at least 20% of death in fewer than five years old children [1].

In Sub-Saharan Africa, the mortality in children under five years old during the period between 1990 and 1998 was doubled compared with the period from 1982 to 1989. One of the most important reasons of this increase in the mortality rates is the spread of antimalarial drug resistance in Africa [2].

The prevalence of malaria infection in children less than five years is 22% and 48% in Malabo (Island Region) and in Bata (Continental Region). The more frequent species in Malabo are *Plasmodium falciparum* (92%), *P. malariae* (3%), *P. ovale* (1%), and mixed infection (4%); in Bata are *Plasmodium falciparum* (97%), *P. malariae* (2%), and mix infection (1%) (unpublished data).

A lot of African countries have the necessity of review its malaria treatment. In Equatorial Guinea, as per the National Malaria Program, chloroquine (CQ) is the first line of treatment, whereas quinine is for treatment of severe malaria. Due to the initiative of Medical Care International Development (MCDI), The Equatorial Guinea Ministry of Health has started to use the combination of AS + SP as a first line treatment of uncomplicated *falciparum* malaria in children and pregnant women since 2004 in Bioko Island.

In different in vivo studies carried out in Equatorial Guinea (Bioko Island) by the Institute of Health Carlos III (Madrid, Spain), in collaboration with the Ministry of Health of this country from 1992 to 1999, it was concluded that resistance to cloroquine (CQ) was over 40% in children under five, and resistance to sulphadoxine-pyrimethamine (SP) was around 25% in the same age group. In the continental area of the country, studies undertaken during 2001 gave the same results as in the island [3].

During the years 2002 and 2003, the sensitivity of *Plasmodium* *falciparum* to AS + SP combination was evaluated in Equatorial Guinea (unpublished study), and an adequate clinical response of 95% was achieved.

As a result of the problem of drug resistance developed in *P. falciparum*, the Word Health Organization (WHO) recommended to improve access of population to a quick and efficacy treatment to decrease the burden of malaria [4]. The current recommendation is the use of a combination therapy following the experience of treatments in other diseases, such as tuberculosis, leprosy, and HIV infection. A combination therapy would be helpful in simultaneous use of two or more blood schizontocidal drugs with independent modes of action and different biochemical targets in the parasite [5].

Present study was undertaken jointly with the Malaria National Control Program for evaluation of AS + SP and AQ + SP combinations in treatment of uncomplicated *falciparum* malaria. AQ + SP combination is presently recommended by the WHO, if the efficacy is acceptable [6]. It is less expensive than a combination with artesunate and can be used as a first line of treatment keeping in view the economic resources of the country rather than introducing directly a combination with artesunate. The second one AS + SP is being used in the Island, and its efficacy needs to be monitored for its further use in the Malaria National Control Program.

For any in vivo study, it is necessary to differentiate recrudescence or reinfection, when the country is having high incidence of malaria and with a high transmission rate. For this reason, polymorphic markers as *msp-1* and *msp-2* or microsatellite markers are useful for this purpose [7].

In this study, we evaluated the efficacy of the AS + SP and AQ + SP combinations as a first-line treatment for uncomplicated malaria in Equatorial Guinea, and the outcome of infection and treatment was determined by molecular analysis to present a real scenario on use of these two combinations.

## 2. Material and Methods

### 2.1. Area and Study Population

Equatorial Guinea is situated in Central Africa, in the Guinea Gulf. It is divided in two regions, the continental area, called Rio Muni between Cameroon and Gabon, and the Island Region (Bioko, Annobón and Corisco Valley). Bioko, the biggest island, is placed 40 km from Cameroon coast. Population estimation is around 484 000 habitants in an area of 28 051 km^2^ between both regions [8]. Around 75% of the population lives in the Continental Region.

Malabo is the capital of the country and is placed in the Island of Bioko. There is a tropical climate with a rainy season from May to October and a dry period from December to March. It is a mesoendemic transmission area with a Plasmodium index of 21.7% in 2005 [9]. Bata is the most important city in the continental region and has also a tropical climate with two dry seasons (December–March, June–September) and two rainy seasons (March–June, September–December) alternate. It is a mesohyperendemic area with a Plasmodium index between 41% and 75% in children under five years old. (Unpublished study from a National Prevalence Survey 2006, Ministry of Health and Instituto de Salud Carlos III.)

In December 2005, an in vivo study was carried out in the two Regional Hospitals of Malabo and Bata. The study followed the WHO methodology [10]. The cases that were included in the assessment came from the paediatric external consultation.

It was a two-armed prospective evaluation of the clinical and parasitological responses to different antimalarial combinations, as AS + SP and AQ + SP. The minimal sample size was different depending on the combinations. In order to have a confidence level of 95% and a precision of 10% and as the proportion of treatment failure with AS + SP was already known, a minimum of 50 patients had to be included. With AQ + SP, there was no evidence of treatment failure, so the minimum sample size had to be 96 for having a precision of 10% with a confidence level of 95% [10].

### 2.2. Procedures, Treatment and Followup

All the children were medically screened. The inclusion criteria were age between 6 to 59 months, no signs of severe malnutrition, a slide confirmed monoespecific infection of *P*. *falciparum,* parasite density between 2000 and 200 000 asexual parasites/microlitre, axillary temperature >37.5°C, easy access to the hospital, absence of history of hypersensitivity reactions to any of the drugs being evaluated and informed consent provided by parents or caregivers. Any danger signs of severe malaria such as inability to drink or breastfeed, vomiting, recent history of convulsions, lethargy, and inability to sit or stand up were considered as exclusion criteria.

All the children were randomly assigned to one therapeutic arm. They were evaluated clinically and parasitologically during 14 days; treatment was given during the first three days under direct observation.

A record form, which included: age, sex, address, name of the caregiver, contact telephone (if available), was maintained. During the followup, parameters such as temperature (during the six days), doses of drug (days 0, 1, and 2), parasitaemia (days 0, 1, 2, 3, 7, and 14 or whatever day that children was bringing to the hospital), haematocrit (days 0 and 14) and filter paper (days 0, 14, or any day in case of treatment failure for analysis of the molecular markers) were recorded. Finger prick blood samples that were collected in Whatman paper and in a thick-and-thin smear stained with Giemsa for microscopy exam were undertaken.

Parasitaemia was quantified by a standard approximation method, that is, number of asexual parasites seen per 200 white blood cells in a high-power examination of a thick blood film. A positive smear was defined as the presence of at least one asexual parasite seen after examining 100 thick smear fields. Quality control of blood smears was done by rereading 10% of slides selected randomly. Discordant results were subjected to a third microscopist. Haematocrit was measured by microhaematocrit centrifugation on days 0 and 14.

Drugs used were amodiaquine 150 mg (Holden Medical, set. 04L02. Expired 11/2007), artesunate 50 mg (Action medeor. Lot. 055585. Expired 11/2008), sulphadoxine 500 mg + pyrimethamine 25 mg (IDA. Lot. SPF-200 Expired 11/2008), quinine 200 mg (Holden Medical. Lot MFF 371/05 A 01. Expired 12/2007).

Amodiaquine was given as 10 mg/kg of body weight per day for three days. Artesunate given as 4 mg/kg of body weight per day for three days and sulphadoxine-pyrimethamine given as sulphadoxine 500 mg + pyrimethamine 25 mg/kg of body weight in a single dose for the first day. In case of treatment failure, quinine at 10 mg/kg of body weight every 8 hours for seven days was administered.

### 2.3. Definition of Treatment Failure

The efficacy outcome was measured based on parasitological clearance rates on day 14. The criteria to determine the treatment failure were the following: early treatment failure (ETF) was considered if development of severe malaria or danger signs during days 1, 2, or 3 in presence of parasitaemia, or parasitaemia on day 2 is higher than day 0 irrespective of axillary temperature, or parasitaemia on day 3 with axillary temperature >37.5°C, or parasitaemia on day 3 more than 25% of day 0.

Late clinical failure (LCF) was considered by development of severe malaria or danger signs after day 3 with parasitaemia without previous criteria of ETF, or parasitaemia and temperature >37.5°C on any day between 4 and 14 without previous criteria of ETF. Late parasitological failure (LPF) was determined based on the presence of parasitaemia between days 7 and 14 with temperature >37.5°C, without previous criteria of ETF or LCF. Adequate clinical and parasitological response was determined (ACPR) by the absence of parasitaemia on day 14, irrespective of axillary temperature, without meeting the criteria of ETF, LCF, and LPF.

Some of the cases were considered as Withdrawal, when caregivers of children decided not to continue with the study despite all efforts and lost to follow up; children could not be found at hospital, in the community, or in the study area.

### 2.4. DNA Extraction and Molecular Study

We extracted DNA from individual blood sample collected on Whatman filter paper. We cut a circle of paper (40 mm Ø) with blood, and DNA was extracted by Phenol/Chloroform method. This DNA was used for the different molecular assays: (a) semi-nested-Multiplex PCR for the diagnosis of Malaria to confirm the *Plasmodium* species (b) study of the *msp-1* and *msp-2* genes of *P. falciparum* through nested-PCR [7]. In order to distinguish recrudescence from reinfection cases, we studied the three different allelic families of *msp-*1 gene: Mad20, RO33 and K1, and *msp-2* gene. Recrudescence was defined as the same population of parasites appeared on day 7 or day 14 as in day 0. Reinfection was defined as a new population of parasites detected on day 14 compared to day 0. After the PCR, we analysed the result in the Bioanalyzer 2100 expert (Agilent Technologies), this method has a very high resolution and we can distinguish amplification fragments with a little difference in the basepairs.

### 2.5. Statistical Analysis

Baseline characteristics, means, and percentages were compared in study subjects of both areas by Student *t*-test and by chi-square test respectively. The non-parametric alternative for *t*-test and Mann-Whitney test was used to compare the baseline parasite density. Therapeutic efficacy was calculated by dividing the number of ACPR cases by the number of patients evaluated on 14th day. The 95% confidence intervals for drug efficacy were also calculated. Log-rank test for equality of survivor functions was calculated in order to compare time to parasitological clearance.

## 3. Results

Between August and December 2005, 206 children were randomised from the external consultation of Malabo and Bata Regional Hospitals in order to evaluate the efficacy of two antimalarial combinations: AQ + SP and AS + SP. Baseline characteristics of children are shown in Table 1. As expected, no differences were found between groups, except in the weight.

**Figure 1:**
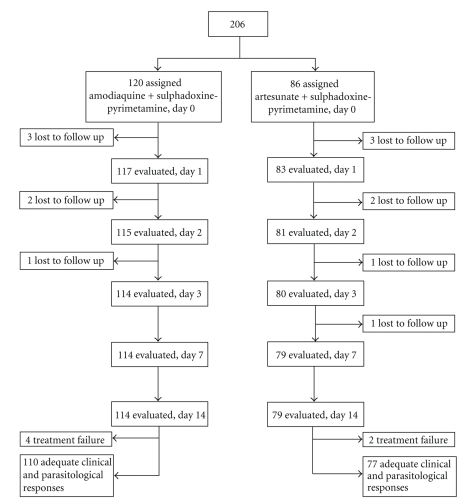
Trial profile.

The enrolled children were given one of the two combinations in a random way: 120 were treated with AQ + SP and 86 received AS + SP. In the first group 95% (114/120) finished the study and 91.9% (79/86) in the second group. The trial profile appears in the Figure 1.

There were 13 children who lost to follow up in AQ + SP group. The percentage of lost to follow up was 5% (6/120) and in the AS + SP arm was 8.1% (7/86). In general children who lost to follow up were fewer (6.3%; 13/206) than 10% of the children as per WHO recommendations.

With AQ + SP, 4 cases of LPF were registered and the proportion of treatment failure was 3.5% (4/114). The ACPR was 96.5% (confidence interval 95%: [91.3%–99%]). With AS + SP, 2 cases of LPF 2.5% (2/79) were registered, the cure rate was 97.5% (C.I. 95%: [91.2%–99.7%]). All of the failure cases received rescue treatment with oral quinine and cured (see Table 2). After the molecular correction, the ACPR changed from 96.5% to 97.3% with AQ + SP combination.

Both combinations were well tolerated, no deaths were notified and no severe reactions were recorded, except 3 patients (2.5%) had complaints of itching in AQ + SP treatment group, they were given antihistamine. Four patients (3.3%) in AQ + SP and 1 patient (1.2%) in AS + SP treatment groups vomited, which could not be confirmed whether this was the consequence of malaria or the treatment effect.

**Figure 2:**
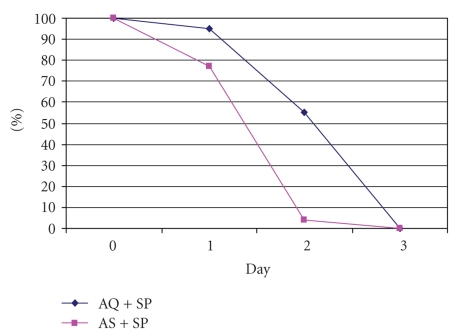
Proportion of parasites clearance with the two combinations.

The *parasite clearance* (Figure 2) was faster in the group who received the combination with artesunate (log-rank test *P* < .001). In AS + SP treatment group, 77.2% (61/79) and in AQ + SP treatment group, 94.7% (108/114) presented parasitemia on day 1. On day 2, 3.8% (3/79) of AS + SP and 55.3% (63/114) of AQ + SP treatment groups were parasitemic. By day 3, all of them were negative.

Baseline *gametocyte carriage* was less than 5% in both arms. After receiving treatment, gametocyte density was found to be higher in AS + SP patients than AQ + SP. With the first combination, by day 2, 12% of children had gametocytes on the thick film, compared with the 7.5% with AQ + SP. By day 3, the 7.8% with AS + SP and the 6% with AQ + SP had gametocytes. By day 14, gametocytemia was under 2% with both the combinations.

*Haematocrit* at enrolment showed that 37% of the children had moderate anaemia with haematocrit 24–29, 49% had mild anaemia with haematocrit 30–34, and 14% were nonanaemic. After treatment the moderate anaemia decreased from 37% to 23%, and nonanaemic cases increased from 14% to 22%.

*Molecular correction* was done to distinguish recrudescent from reinfection in 6 treatment failure cases: 4 cases from Bata, 3 in the AQ + SP combination, and 1 in the AS + SP, and 2 in Malabo with two combinations (AQ + SP and AS + SP). Table 3 shows the number of alleles of *msp-1* allelic family, and *msp-2* detected in the six treatment failure cases. We could confirm the resistant cases from Bata by molecular analysis; because the parasite genotypes detected by PCR on day 14 were similar with those on day 0. However, in the AQ + SP treatment failure cases from Malabo, one of them was reinfection because a different set of genotypes appeared on day 14 as shown in Table 3.

## 4. Discussion

There is a need to give a thought about any monotherapy as a first-line treatment in case of patients report with uncomplicated *falciparum* malaria. Keeping this in view, present study was undertaken on the drug sensitivity of two different therapeutics combinations. This study would be helpful in National Malaria Control Program of Equatorial Guinea to introduce a new combination therapy for malaria treatment.

Two combinations were assessed, the combinations AS + SP, where the AS is an efficacious drug and reduces the parasitaemia faster than others, and the second combination, AQ + SP, is also an effective combination but with the risk to develop resistant in due course of time, but more accessible to the population when compared with artemisinin derivatives.

The study demonstrated that both combinations are safe and efficacious for the treatment of nonsevere *falciparum* malaria in Equatorial Guinea. These results are similar to other studies made in different African's areas. A study carried out in Tanzania in an area of high malaria transmission compared the efficacy and safety of AQ and SP in monotherapy and AQ + SP as a combination. The study demonstrated that AQ + SP combination was safe, and the combination of the two drugs had higher efficacy (96.2%) than monotherapy [11]. In a similar study carried out in Gambian children, concluded that AS + SP combination was safe and the efficacy was 98.4% [12]. A study carried out in Guinea showed an efficacy of 99% with this combination [13]. These results are similar to those of the present study with cure rates of 97.5% (77/79) in AS + SP combination and 97.3% (111/114) in AQ + SP. The situation in Equatorial Guinea is similar with other African countries. The cure-rates with the two combination drugs, AQ + SP and AS + SP, are over the 95%, which is the limit that WHO suggested for a combination to be used as a first line of treatment [6].

In general terms, drugs were well tolerated, with a low percentage of adverse effects. In the group that received AQ + SP, a small number of children required retreatment after vomiting after treatment. Severe reactions with amodiaquine as neutropenia have been described also in other publications. In one study, it was shown that the risk was associated with amodiaquine used as a weekly prophylaxis, not like a treatment [14]. In a study carried out in Madagascar, using combination of AQ + SP like our work, no severe reaction was described using AQ in a 3-day treatment schedule [15].

Clearance of parasites was faster in case of combination with artemisinin derivatives, due to the mode of action of artemisine, which reduces parasitaemia by a factor of 10 in each cycle. In Uganda, was described the difference in parasitemia clearance using a combination with and without artemisinin derivatives. By day two, in the first case just 3% were positive compared to the second group, where 48% were positive [16]. These results coincide with our study, where by day two, 3.8% were parasitaemic compared with the 55.3% of children who received AQ + SP on the same day. Due to faster parasite clearance, clinical improvement and disappearance of symptoms were faster in children treated with AS + SP. Because of this rapid effect, use of artemisinin derivatives in a combination would be appropriate in terms of decreasing the possible development of parasite resistance to the drug by rapid clearance thus they are not in contact with the drug for a long time [17].

Contrary to other studies, we have not seen any difference in gametocytes carriage. From this study, we could not say that using of artemisinin-based combination therapies may reflect in decrease of malaria transmission in the area. One study in Tanzania, where different drugs in monotherapy and combinations were compared, demonstrated that AQ + SP gametocytes on day 14 were 25.7% in children, which was double than using the combination with AS, where just 11.9% of children had gametocytes [18].

Both the combinations had SP. It is necessary to assess other therapies without this drug due to the following reasons. First, SP is the only safe drug actually to be used as Intermittent Preventive Treatment for pregnant women. Following WHO recommendations, we need to protect the future development of resistance to this drug. And the second reason is that SP has a resistance around 18% in monotherapy (unpublished data, 2002). Drugs that have been used for a long time as monotherapy and have already demonstrated resistance have the risk to develop resistance in combination in due course of time.

From the results of this study, we may suggest that in all in vivo studies a molecular assay for discriminating recrudescence from reinfection should be done. The molecular correction is the only method that allows us to give a real data about the level of resistance detected in an endemic area. This way, it is possible to analyse different markers like *m* *s* *p*-1 and *m* *s* *p*-2 genes of *P. falciparum*, or other polymorphic markers as microsatellites [19].

We need to continue the epidemiological surveillance and monitoring resistance in order to give enough information to the National Malaria Program of Equatorial Guinea for revising malaria treatment policy in the country. This should be done as per the recommendation of WHO before the drug resistance reaches at levels not allowed to be used.

The conclusions of this study are that both the combinations are safe and efficacious for their use as a first-line treatment in case of nonsevere *falciparum* malaria. To choose one of them will depend on economic resources of Equatorial Guinea in the moment to change Malaria Policy of the country; the molecular correction gives complementary information to strengthen the findings of in vivo studies carried out in areas with a high level of transmission.

## Figures and Tables

**Table 1:** Baseline characteristics.

	Amodiaquine + sulphadoxine-pyrimethamine	Artesunate + sulphadoxine-pyrimethamine
Sex		
Male (%)	62	53
Age in months (mean ± sd)	28.2 (15)	26.7 (15)
Weight in Kg (mean ± sd)*	12.7 (4)	11.7 (3)
Temperature in ºC (mean ± sd)	38.2 (1)	38.3 (1)
Parasites/microlitre (median/IR)	23,882 (9,360–30,480)	24,978 (20,040–31,640)
Haematocrit	30 (4)	31 (4)

*P < .05; $*P* < .01; sd (standard deviation); IR (Interquartile rank).

**Table 2:** Treatment outcomes observed in the patients of Bata and Malabo Hospitals. The results appear in the different groups with two combination drugs.

Treatment	Total	AQPR		ETF		LPF		LCF	
		Nº	%	Nº	%	Nº	%	Nº	%
amodiaquine + sulphadoxine-pyrimethamine	114	110	96.5	0	0.0	4	3.5	0	0.0
artesunate + sulphadoxine-pyrimethamine	79	77	97.5	0	0.0	2	2.5	0	0.0

**Table 3:** Number of alleles of *msp-1* allelic family, and the alleles detected in *msp-2*. In case of isolate 293, new populations appeared on day 14, one for RO33 (150 bp) and two for *msp-2* (600 bp and 700 bp).

			*msp*-1			
	Locality	Treatment	K1	MAD20	RO33	*msp-2*
36D0	Bata	AQ/SP	135/155/225	0	0	570/755
36D14			155	0		0
11D0	Bata	AQ/SP	190/205/290		150	500/530/590
11D14						500
102D0	Bata	AQ/SP	210/250/265	195/210/240	150	535/590
102D14					150	
75D0	Bata	AS/SP	160/220/250		150	575/670
75D14					150	
29D0	Malabo	AQ/SP	135/225			490/525/665
29D14					150	600 / 700
2D0	Malabo	AS/SP	210/250/265	195/210/240	150	535/590
2D14			∗	∗	∗	∗

## References

[1] WHO Africa Malaria Report.

[2] WHO Drug Resistance in Malaria.

[3] Roche J, Guerra-Neira A, Raso J, Benito A (2003). Surveillance of in vivo resistance of *Plasmodium falciparum* to antimalarial drugs from 1992 to 1999 in Malabo (Equatorial Guinea). *American Journal of Tropical Medicine and Hygiene*.

[4] WHO The African Summit on Roll Back Malaria.

[5] WHO Antimalarial drug combination therapy.

[6] WHO Guidelines for the treatment of malaria.

[7] Robert F, Ntoumi F, Angel G (1996). Extensive genetic diversity of *Plasmodium falciparum* isolates collected from patients with severe malaria in Dakar, Senegal. *Transactions of the Royal Society of Tropical Medicine and Hygiene*.

[8] UNPD World Population Prospects: The 2006 Division.

[9] Pardo G, Descalzo MA, Molina L (2006). Impact of different strategies to control *Plasmodium* infection and anaemia on the island of Bioko (Equatorial Guinea). *Malaria Journal*.

[10] WHO Assessment and monitoring of antimalarial drug efficacy for the treatment of uncomplicated falciparum malaria.

[11] Schellenberg D, Kahigwa E, Drakeley C (2002). The safety and efficacy of sulfadoxine-pyrimethamine, amodiaquine, and their combination in the treatment of uncomplicated *Plasmodium falciparum* malaria. *American Journal of Tropical Medicine and Hygiene*.

[12] von Seidlein L, Milligan P, Pinder M (2000). Efficacy of artesunate plus pyrimethamine-sulphadoxine for uncomplicated malaria in Gambian children: a double-blind, randomised, controlled trial. *The Lancet*.

[13] Bonnet M, Roper C, Félix M, Coulibaly L, Kankolongo GM, Guthmann JP (2007). Efficacy of antimalarial treatment in Guinea: in vivo study of two artemisinin combination therapies in Dabola and molecular markers of resistance to sulphadoxine-pyrimethamine in N’Zérékoré. *Malaria Journal*.

[14] Phillips-Howard PA, Bjorkman AB (1990). Ascertainment of risk of serious adverse reactions associated with chemoprophylactic antimalarial drugs. *Bulletin of the World Health Organization*.

[15] Ménard D, Andrianina NNH, Ramiandrasoa Z (2007). Randomized clinical trial of artemisinin versus non-artemisinin combination therapy for uncomplicated falciparum malaria in Madagascar. *Malaria Journal*.

[16] Dorsey G, Staedke S, Clark TD (2007). Combination therapy for uncomplicated falciparum malaria in Ugandan children: a randomized trial. *Journal of the American Medical Association*.

[17] White NJ (1998). Preventing antimalarial drug resistance. *Drug Resist Updates*.

[18] Mutabingwa TK, Anthony D, Heller A (2005). Amodiaquine alone, amodiaquine+sulfadoxine-pyrimethamine, amodiaquine+artesunate, and artemether-lumefantrine for outpatient treatment of malaria in Tanzanian children: a four-arm randomised effectiveness trial. *The Lancet*.

[19] Mwangi JM, Omar SA, Ranford-Cartwright LC (2006). Comparison of microsatellite and antigen-coding loci for differentiating recrudescing *Plasmodium falciparum* infections from reinfections in Kenya. *International Journal for Parasitology*.

